# Long-term effect of early-life arsenic exposure on morning plasma cortisol in adults from Antofagasta, Chile

**DOI:** 10.1186/s12940-026-01304-9

**Published:** 2026-05-06

**Authors:** Rosemarie de la Rosa, Craig Steinmaus, Anthony Nardone, Amanda Silveira, Johanna Acevedo, Catterina Ferreccio, Martyn T. Smith, Fenna C. M. Sillé

**Affiliations:** 1 Division of Environmental Health Sciences, School of Public Health, University of California, Berkeley, CA USA; 2 Arsenic Health Effects Research Group, School of Public Health, Room 5302, University of California Berkeley, 2121 Berkeley Way, Berkeley, CA 94720-7360 USA; 3 UC San Francisco (UCSF) Joint Medical Program, UCSF School of Medicine, San Francisco, CA USA; 4 University of California (UC) Berkeley, UC Berkeley School of Public Health, Berkeley, CA USA; 5 Department of Nutritional Sciences and Toxicology, University of California, Berkeley, CA USA; 6 School of Medicine, Pontificia Universidad Católica de Chile, Advanced Center for Chronic Diseases (ACCDiS), Santiago, Chile; 7 Department of Environmental Health and Engineering, Bloomberg School of Public Health, Johns Hopkins University, Baltimore, MD USA

**Keywords:** Arsenic, Early-life, Cumulative, Glucocorticoid, Sex-dependent

## Abstract

Over 100,000 people were exposed to arsenic-contaminated drinking water in Antofagasta, Chile from 1958–1970. Individuals born during this high exposure period have elevated rates of cancer, lung and cardiovascular disease, and hypertension. However, the mechanisms of long-term arsenic toxicity remain unclear. We investigated whether early-life arsenic exposure was associated with altered glucocorticoid levels in adulthood. This study included 114 individuals born in Antofagasta during the high exposure period and 118 individuals born in other Chilean cities with lower exposure. Arsenic exposure metrics were constructed based on residential histories and included: concentration at birth, peak and highest 5-year average between ages 0–10 years, and highest lifetime 5-year average, and lifetime cumulative exposure. Morning plasma cortisol concentrations were measured using a cell-based bioassay. Individuals in the highest quartile of highest lifetime 5-year average of arsenic exposure had approximately 11% lower mean log cortisol levels than those in the lowest quartile of exposure (β = -0.116; 95% CI: -0.229, -0.003). In sex-stratified analyses, associations were stronger among female participants. For example, female participants in the highest quartile of cumulative exposure had 22.0% lower cortisol levels compared to those in the lowest quartile (β = -0.248; 95% CI: -0.444, -0.053) and the test for interaction by sex was statistically significant (p = 0.036). This study is the first to show that early-life arsenic exposure may suppress HPA axis activity decades after exposure has ceased. These findings support endocrine disruption as a potential mechanism underlying long-term health effects of arsenic and highlight early development as a critical exposure window.

**Supplementary Information:**

The online version contains supplementary material available at 10.1186/s12940-026-01304-9.

## Introduction

An estimated 220 million people worldwide are exposed to arsenic-contaminated drinking water at levels that exceed the World Health Organization (WHO) provisional guideline of 10 µg/L [35]. This widespread exposure to arsenic poses a major global public health concern. Inorganic arsenic is a Group 1 human carcinogen and has been associated with a wide range of health effects, including skin lesions, cardiovascular disease, hypertension, diabetes, and adverse developmental outcomes [31, 32]. Furthermore, the developing fetus and children are particularly vulnerable to arsenic exposure, as early-life exposure can have long-term effects that increase the risk of chronic diseases in adulthood [43]. There is an important need to better understand how early-life arsenic exposure leads to long-term health outcomes.

Northern Chile presents a unique opportunity to investigate the long-term health effects of arsenic exposure from drinking water. Antofagasta, one of the largest cities in Northern Chile, had a distinct period of exceptionally high arsenic exposure that began in 1958 when rapid population growth led to supplementation of the city’s water supply with rivers containing arsenic concentrations of 860 μg/L and ended in 1970 with the installation of an arsenic removal treatment plant [40]. As a result, more than 100,000 people were exposed to arsenic concentrations that exceeded the current WHO arsenic standard by greater than 80-fold for a period of 12 years. This exposure scenario, with its distinct start and stop, large population, and good exposure records provides a rare opportunity to investigate the long-term consequences of arsenic exposure. Ecological studies conducted in this region of Chile observed that adults exposed to high levels of arsenic-contaminated drinking water in utero and during early childhood had higher mortality rates from bronchiectasis [46], acute myocardial infarction [57], and cancer [44, 56] compared to those who were lesser exposed or exposed only as adults. In addition to mortality, follow-up studies in this same population observed a higher incidence of lung and bladder cancer [51], impaired lung-function [12, 50] and increased prevalence of hypertension [20] among adults with high in utero and early-life arsenic exposure, with many associations persisting 30–40 years after the high exposure period ended [39]. Collectively, these studies provide strong evidence that early-life arsenic exposure induces persistent alterations that promote disease into adulthood. Understanding mechanisms of these persistent effects is crucial to inform public health strategies to mitigate the long-term health effects of early-life arsenic exposure.

Several biological mechanisms have been hypothesized to underly the persistent effects of early-life arsenic exposure, including altered epigenetic reprogramming, immune modulation, and oxidative stress [5, 53]. These mechanisms may also contribute to the endocrine-disrupting effects of arsenic on the hypothalamic–pituitary–adrenal (HPA) axis, a highly conserved neuroendocrine system that regulates the body’s response to stressors and maintains metabolic and immune homeostasis through the secretion of glucocorticoids (cortisol in humans and corticosterone in rodents) [52]. Dysregulation of the HPA axis can follow a biphasic pattern, with initial over-activation leading to hypercortisolism, followed by chronic impairment of negative feedback mechanisms that result in hypocortisolism [38]. Both extremes of cortisol dysregulation contribute to pathologies similar to those observed among arsenic-exposed individuals, including metabolic dysfunction, hypertension, inflammation, and immunosuppression [37, 54]. Animal studies have demonstrated that prenatal arsenic exposure induces long-term HPA axis dysfunction that persists into adulthood, characterized by elevated basal corticosteroid levels, blunted stress reactivity, and impaired negative feedback in part through altered expression of genes governing glucocorticoid signaling and metabolism (e.g., *NR3C1,11β-HSD1/2*) [8, 17, 26]. The effects of early-life arsenic exposure on the HPA axis also appear to differ by sex, with male mice being more susceptible to these effects [1, 47]. Further research is needed to better understand the molecular and sex-specific effects of early-life arsenic exposure on the HPA axis in humans.

Epidemiological studies examining the effects of arsenic exposure on plasma cortisol are limited and have yielded inconsistent findings. A study of 267 women in West Bengal, India observed that individuals currently exposed to 11–50 μg/L of arsenic in drinking water had nearly two-fold higher plasma cortisol levels than those exposed to < 10 μg/L arsenic, although the association was not statistically significant [42]. In contrast, a longitudinal study of 168 mother–child dyads in Arica, Chile found that higher maternal second-trimester urinary arsenic concentrations were associated with lower salivary cortisol in their infants 18–24 months after delivery [41]. Null associations have also been reported in studies where co-exposure to other heavy metals was present [10, 25]. These inconsistent findings may reflect variability in exposure characteristics (e.g., timing, concentrations, and sources), as well as differences in how both exposure and cortisol were measured. It should be noted that most studies to date have been cross-sectional and the only longitudinal study of prenatal arsenic exposure did not assess cortisol levels beyond infancy [41]. Additional longitudinal studies are needed to determine whether early-life arsenic exposure influences cortisol regulation beyond this developmental period and across the lifecourse.

Here, we investigated associations between early-life and lifetime arsenic exposure with morning plasma cortisol concentrations in adulthood. Based on evidence from human and animal studies, we hypothesized that early life arsenic exposure would be associated with altered morning cortisol concentrations, suggesting perturbed HPA axis regulation. This study included 233 adults currently living in Antofagasta, with 114 individuals born in Antofagasta during the high exposure period and 118 individuals who were born in other Chilean cities with lower levels of exposure. Since early-life arsenic exposure has been shown to have sex-specific effects on HPA axis activity in animal studies, we also explored sex differences in the association between arsenic exposure and plasma cortisol.

## Methods

### Study participants

We leveraged data and biospecimens collected from a study originally designed to examine the long-term effects of early-life arsenic exposure on immune function. Participants were a convenience sample of employees from the Antofagasta Hospital and University of Antofagasta as well as participants from general recruitment across Antofagasta in Chile. Recruitment occurred during two time periods: November 2013-January 2014 and May–July 2017. The study included 114 participants who were born in Antofagasta during the high exposure period (1958–1970) and who still resided in Antofagasta at the time of recruitment along with a comparison group of 118 participants that were born in other cities throughout Chile with lower exposure and moved to Antofagasta after the high exposure period. Given the original focus on immune function, study exclusion criteria included antibiotic use in the past 3 months, use of enemas or laxatives more than once per month, or use of steroids or immunosuppressants. Out of the 250 individuals initially recruited for the study, only those born during the high exposure period (1958–1970) were included in this analysis (n = 233). Consistent with the 1958–1970 exposure window, participant ages at recruitment were 43–55 years for the 2013 study and 47–59 years for the 2017 study. We also excluded one participant that had plasma cortisol levels below the assay’s limit of detection (described in Sect. "Plasma Cortisol Equivalent Concentrations"), resulting in a final sample of 232 participants.

### Arsenic exposure

The combination of relatively few water sources due to the arid climate and availability of historical records allows for the construction of accurate lifetime arsenic exposure estimates for individuals, from birth through adulthood, simply by knowing the cities in which they have lived. Therefore, we collected a detailed lifetime residential history from each participant using a standardized questionnaire. Participants were asked to provide all residences where they lived for ≥ 6 months, the water source at each residence (e.g. bottled water, tap), and their typical daily water intake currently as well as 20 and 40 years ago. We then linked each participant’s city of residence to annual arsenic water measurements obtained from government agencies, water companies, and other sources that were available for all large cities and towns in Chile [15]. While arsenic levels outside of Antofagasta were generally below 10 µg/L [45], it is important to note that some nearby towns also had elevated arsenic levels in their drinking water supply. While not as high as Antofagasta, arsenic concentrations at birth for 47 of 118 participants born in other cities between 1958–1970 still exceeded Chile’s national drinking water standard of 50 µg/L (this standard was later reduced to 10 µg/L in 2005). Therefore, we also calculated several arsenic exposure metrics for each participant to assess both the intensity and cumulative nature of exposure. These individual-level arsenic exposure metrics included: born in Antofagasta during the high exposure period (1958–1970) as a binary variable (no/yes); arsenic concentration in drinking water in their birth city; peak exposure during 0–10 years of life to capture the highest single annual concentration during early-life; and highest 5-year average exposure during 0–10 years of life to assess sustained high exposure during early-life. The 0–10 year age range was selected because most participants' cumulative arsenic exposure occurred during their first decade of life (Supplemental Fig. 2). Lifetime highest 5-year average exposure was also estimated as the highest annual concentration averaged over any contiguous 5-year period. Lastly, lifetime cumulative arsenic exposure was calculated for each participant by summing yearly concentrations from birth until the time of sample collection.

### Plasma cortisol equivalent concentrations

A single fasted blood sample was collected in EDTA tubes from each willing participant and time of sample collection was recorded by research personnel to adjust for the diurnal nature of cortisol secretion in statistical analyses. The average time of blood draw was 9:00AM and ranged from 6:10AM to 12:35PM. All samples were processed within 8 h upon collection and plasma aliquots were frozen at −80˚C for 2–8 weeks before being transported on dry ice to the University of California, Berkeley where they were stored until analysis. Cortisol assays were performed in two separate batches. Cortisol assays for samples collected during the 2013 and 2017 recruitment periods were performed three years and one year after collection, respectively.

Plasma cortisol equivalent concentration values were obtained using the 231GRE cell-based bioassay developed by our research group [13]. We previously demonstrated high concordance correlation (r_c_ = 0.92) between this assay and cortisol enzyme-linked immunosorbent assay measurements in plasma collected from 12 healthy individuals. Briefly, the 231GRE cell line was generated by stably transfecting MDA-MB-231 cells with a luciferase reporter gene driven by three simple glucocorticoid-response elements. These cells were maintained at 37 °C with 5% CO_2_ using Dulbecco’s Modified Eagle Medium (DMEM; Gibco) containing 10% fetal bovine serum (FBS; Atlanta Biologicals). One week prior to experiments, cells were cultured using phenol red-free DMEM (Hyclone) with 10% charcoal–dextran FBS (Atlanta Biological) to reduce the concentration of hormones present in media. Hormone-stripped cells were seeded at 2.7 × 10^4^ cells/well in white 96-well plates and incubated at 37 °C overnight to allow cells to attach. The next day, media in quadruplicate wells was replaced with 100 µL of media containing either a range of cortisol standards (0, 1.56, 3.13, 6.25, 12.5, 25 nM) or human plasma samples diluted 1:20 in hormone-depleted media. Cells were treated for 24 h at 37 °C before rinsing wells with PBS and adding 1 × cell lysis buffer (Promega). Luminescence was quantified as relative light units (RLUs), which are proportional to the degree of luciferase reporter activity, using a Berthold Centro XS3 LB 960 microplate luminometer and an automatic injector for Luciferase Assay Reagent (Promega). Duplicate cortisol standards (0–25 nM in 0.1% DMSO) and a common reference plasma sample (diluted 1:20 run in quadruplicate) were included on every plate.

RLUs reflect the total amount of glucocorticogenic compounds present in media containing diluted plasma samples. Wells with RLUs below the lowest cortisol standard on the plate (1.56 nM) were excluded from the analysis to avoid bias from imputing values below this concentration. This resulted in one participant being dropped from the analysis. Sample RLUs were converted to cortisol equivalent concentrations by interpolating values from standard curves on each plate that were fit with an inverse weighted 4-parameter logistic regression model. The standard curves for cortisol on each plate fit well with a median R^2^ value of 0.994 (IQR: 0.991, 0.997) across all 26 plates. To minimize plate-to-plate variability and account for batch effects from running the 2013 and 2017 studies separately, cortisol equivalent concentration values were normalized using a common reference plasma sample included on each plate. Estimated cortisol concentrations for each well on a plate were divided by the median concentration of the reference sample on its respective plate and then multiplied by the overall median concentration of the reference sample across all plates. Plate and batch effects were assessed both before and after normalization with the reference sample. After normalization, neither effect remained statistically significant. These normalized cortisol equivalent concentration values were then averaged for each participant and multiplied by 20 to account for the plasma dilution factor. Assay CVs for all participant samples ranged from 0.6% to 23.8% with a mean of 6.3%. Intra-assay CVs for the 2013 and 2017 batches were comparable (Supplemental Table 1). 

### Other covariate data

Covariates were selected based on existing literature examining associations between heavy metals and metalloids (e.g. arsenic and lead) with cortisol levels [11, 19, 34, 41, 48]. Participants age (years), sex (male vs. female), smoking status (ever vs. never), and highest education or grade achieved (< secondary school vs. ≥ secondary school) were assessed via questionnaire. Recruitment period was included as a covariate in our analyses since there were significant differences in arsenic exposure levels between participants recruited in 2013 and 2017 (Supplemental Table 2). Blood sample collection time was also included as hours since 6AM and modeled as a spline with 3 degrees of freedom to account for the diurnal rhythm of cortisol (Supplemental Fig. 1).

### Statistical analysis

Sociodemographic characteristics were compared between participants born in Antofagasta and those born in cities with lower arsenic levels in drinking water using Fisher’s exact test for categorical variables and Wilcoxon rank sum test for continuous variables. Given the exposure range and non-linear relationship with cortisol [41], arsenic concentration at birth was analyzed by tertiles with the lowest concentration group serving as the reference. The remaining arsenic exposure metrics were assessed using quantiles. Plasma cortisol equivalent concentrations were log-transformed since its distribution was right-skewed. Unadjusted linear regression analyses were used to evaluate univariate associations between sociodemographic factors and plasma cortisol equivalent concentrations. Multivariable regression models were used to estimate mean differences in plasma cortisol equivalent concentration by arsenic exposure group. Final models were adjusted for age, sex, smoking status, education level, time of blood sample collection, and study recruitment period (2013 vs. 2017). Based on evidence from animal studies demonstrating sex-specific effects [1, 8, 47], analyses between arsenic exposure groups and log-transformed cortisol equivalent concentrations were stratified by sex. A multiplicative interaction term between arsenic exposure and sex was also included to test for potential effect modification. Analyses were also stratified by recruitment period and age (above or below the median age of 52 years) to rule out potential confounding by these variables. Percent differences in cortisol concentrations were calculated as [e^β^ – 1] × 100, where β is the coefficient from log-transformed models. All p-values were two-sided and a value < 0.05 was considered statistically significant. Statistical analyses were conducted using R software, version 4.3.1.

## Results

### Study population sociodemographic characteristics

**Fig. 1:**
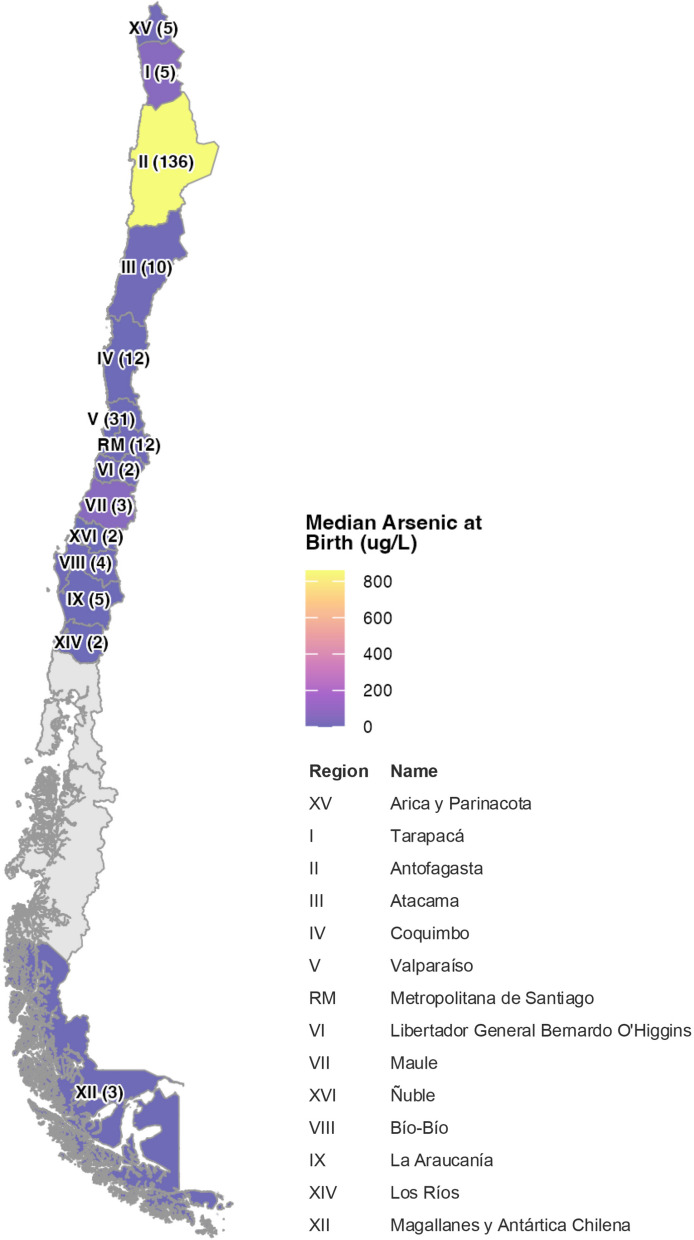
Map of participants birth region. The color gradient represents median arsenic concentration at birth among participants born in each region with the number of participants shown in parentheses next to the region number on map. The Antofagasta region includes 114 participants born in the city of Antofagasta and 22 participants born in other cities within the region, including Chuquicamata, Maria Elena, Pedro de Valdivia, and Tocopilla

Of the total 232 study participants, 114 (49.1%) were born in the city of Antofagasta during the high arsenic exposure period and 118 (50.9%) were born in other regions of Chile with lower arsenic exposure (Fig. 1). Table 1 summarizes the sociodemographic characteristics for all participants as well as stratified by birthplace (Antofagasta vs. other Chilean cities). Participants born in Antofagasta were comparable in age (median: 52 vs 51 years) and sex (54.4% female vs 55.9% male participants) to those born in other Chilean cities. The two groups were also similar in the proportion of participants from each recruitment period and their blood collection times. However, there were slightly fewer individuals who reported ever smoking (52.6% vs 66.1%) and completing secondary school (75.4% vs 84.7%) among participants born in Antofagasta compared to the those born in other Chilean cities. All arsenic exposure metrics, except for lifetime average after age 20, were higher among participants born in Antofagasta. The median concentration of arsenic in drinking water at birth was 860 μg/L for participants born in Antofagasta and 6.2 μg/L for those born in other Chilean cities with lower arsenic levels in drinking water. In contrast, lifetime average concentration levels after age 20 were comparable between the two exposure groups (median: 26 vs 24 μg/L).

**Table 1:** Characteristics of study population

	All (*N* = 232)	Antofagasta (*N *= 114)	Other Chilean Cities (*N* = 118)	*p*-value
Age (years), median (IQR)	52 (48, 55)	52 (48, 55.8)	51 (48, 55)	0.10
Sex, n (%)				0.90
Male	128 (55.2)	65 (54.4)	66 (55.9)	
Female	104 (44.8)	52 (45.6)	52 (44.1)	
Smoking Status, n (%)				0.04
Never	94 (40.5)	54 (47.4)	40 (33.9)	
Ever	138 (59.5)	60 (52.6)	78 (66.1)	
Education, n (%)				0.09
Completed secondary or higher	186 (80.2)	86 (75.4)	100 (84.7)	
Less than secondary	44 (19.0)	27 (23.7)	17 (14.4)	
Missing	2 (0.9)	1 (0.9)	1 (0.8)	
Recruitment Year, n (%)				0.17
2013	84 (36.2)	36 (31.6)	48 (40.7)	
2017	148 (63.8)	78 (68.4)	70 (59.3)	
Blood Collection Time (AM), median (IQR)	8:50 (8:20, 9:50)	9:00 (8:30, 9:50)	8:50 (8:15, 9:50)	0.62
Arsenic Exposure Metrics, median (IQR)				
Water concentration at birth (μg/L)	250 (3.5, 860)	860 (860, 860)	6.2 (0, 150)	<0.001
Peak exposure from birth to 10 years (µg/L)	505.5 (10, 860)	688 (516, 860)	110 (0.5, 510.8)	<0.001
Highest 5-year average from birth to 10 years (µg/L)	300 (10, 688)	688 (297.2, 860)	60 (0.2, 318)	<0.001
Lifetime highest 5-year average (μg/L)	300 (63, 688)	688 (287, 860)	110 (32.2, 334.5)	<0.001
Lifetime cumulative (μg/L-yr)	4013 (1304.6, 7553.8)	6502.2 (3328.6, 9334.3)	1898.5 (539.1, 4553.0)	<0.001
Lifetime average after age 20 (μg/L)	25.3 (17.4, 30.4)	26.0 (19.7, 31.0)	24.0 (12.0, 29.8)	0.01

When comparing demographics between the 2013 and 2017 recruitment periods, the latter tended to be older (median age 48 vs 54 years), included more female participants (35.7% vs. 50.0%), ever smokers (66.7% vs 55.4%), and had less participants who completed secondary school (88.1% vs 75.7%) than in 2013 (Supplemental Table 2). Cumulative arsenic exposure levels were also greater among participants in the 2017 study.

### Associations between sociodemographic characteristics and cortisol

Table 2 lists associations between sociodemographic factors and log-transformed morning plasma cortisol concentration values. The median log cortisol concentration among participants was 5.1 nM (range: 4.3–6.0 nM). Blood collection time was inversely associated with cortisol concentrations, such that values declined the later the blood was collected. Mean plasma cortisol concentrations were ~ 15% lower among female participants compared to male participants (ß = −0.162; 95% CI: −0.239, −0.085). Cortisol concentrations did not differ by age, education, smoking status, or year of recruitment.

**Table 2:** Associations between sociodemographic factors and plasma cortisol equivalent concentrations

**Variable**	**ln(Cortisol) (nM),** **Median (IQR)**	**ß (95% CI)**
All	5.11 (4.88, 5.34)	
Continuous		
Age (years)		−0.005 (−0.014, 0.004)
Blood Collection Time (hours since 6AM)		−0.070 (−0.106, −0.036)
Categorical		
Sex		
Male	5.20 (4.98, 5.38)	Ref
Female	5.01 (4.82, 5.24)	−0.162 (−0.239, −0.085)
Smoking status		
Never	5.07 (4.84, 5.28)	Ref
Ever	5.14 (4.92, 5.37)	0.067 (−0.013, 0.148)
Education		
Completed secondary or higher	5.11 (4.88, 5.31)	Ref
Less than secondary	5.13 (4.86, 5.37)	0.003 (−0.098, 0.104)
Recruitment year		
2013	5.17 (4.93, 5.37)	Ref
2017	5.07 (4.86, 5.29)	−0.067 (−0.149, 0.015)

### Associations between arsenic exposure and cortisol

Associations between arsenic exposure and morning plasma cortisol concentrations were examined using linear regression (Table 3). In unadjusted models, cortisol concentrations did not differ between individuals born in Antofagasta and those born in other Chilean cities with lower arsenic levels in drinking water. Cortisol concentrations were similar between individuals in the highest and lowest tertile of arsenic concentration at birth. For peak arsenic exposure and highest 5-year average exposure during ages 0–10 years, individuals in the highest exposure quartile (peak: 860 ug/L; highest 5 year: >688 ug/L) had approximately 11% lower mean cortisol concentrations compared to those in the lowest exposure quartile (ß _peak exposure 0-10y=860 μg/L_ = −0.117; 95% CI: −0.225, −0.010; ß _highest 5-year average 0-10y>688_ = −0.115; 95% CI: −0.227, −0.004). Cortisol concentrations were lower across all quartiles of highest 5-year average exposure above the lowest quartile (≤ 63 μg/L). For cumulative lifetime arsenic exposure, lower cortisol concentrations were observed for the second (ß = −0.192; 95% CI: −0.302, −0.082) and fourth (ß = −0.119; 95% CI: −0.229, −0.009) quartiles relative to the first exposure quartile. Adjusting models for covariates modestly attenuated effect sizes. Consequently, only associations with lifetime highest 5-year average exposure and the second quartile of cumulative lifetime exposure remained statistically significant after covariate adjustment.

**Table 3:** Associations between arsenic exposure metrics and plasma cortisol equivalent concentrations

**Arsenic exposure metrics**	**N**	**Unadjusted** **ß (95% CI)**	**Adjusted**^**1**^ **ß (95% CI)**
Born in Antofagasta			
No	118	Ref	Ref
Yes	114	−0.036 (−0.115, 0.043)	−0.019 (−0.095, 0.056)
Concentration at birth (μg/L)			
≤ 10	79	Ref	Ref
11–859	53	−0.063 (−0.170, 0.044)	−0.052 (−0.152, 0.049)
860	100	−0.039 (−0.130, 0.052)	−0.036 (−0.123, 0.050)
Peak Exposure 0–10 years (μg/L)			
≤ 10	61	Ref	Ref
11–516	58	−0.093 (−0.203, 0.017)	−0.069 (−0.172, 0.034)
517–859	48	−0.069 (−0.185, 0.046)	−0.061 (−0.173, 0.051)
860	65	−0.117 (−0.225, −0.010)	−0.095 (−0.196, 0.007)
Highest 5-year average from 0–10 years (μg/L)			
≤ 10	61	Ref	Ref
11–300	56	−0.100 (−0.212, 0.011)	−0.081 (−0.185, 0.024)
301–688	60	−0.073 (−0.182, 0.036)	−0.058 (−0.163, 0.047)
> 688	55	−0.115 (−0.227, −0.004)	−0.093 (−0.202, 0.016)
Lifetime highest 5-year average (μg/L)			
≤ 63	58	Ref	Ref
64–300	59	−0.160 (−0.270, −0.050)	−0.124 (−0.229, −0.020)
301–688	62	−0.132 (−0.241, −0.024)	−0.119 (−0.225, −0.013)
> 688	53	−0.142 (−0.255, −0.029)	−0.116 (−0.229, −0.003)
Lifetime cumulative (μg/L-yr)			
≤ 1300	58	Ref	
1301-4010	58	−0.192 (−0.302, −0.082)	−0.159 (−0.267, −0.052)
4011-7550	58	−0.101 (−0.211, 0.009)	−0.061 (−0.167, 0.045)
> 7550	58	−0.119 (−0.229, −0.009)	−0.109 (−0.223, 0.009)

^1^Model is adjusted for age (continuous), sex, education (< secondary vs. ≥ secondary), smoking status (ever vs. never), recruitment year, and time of blood collection (hours since 6AM, modeled as a spline with 3 degrees of freedom)

### Stratified analyses by sex, age, and recruitment period

**Fig. 2:**
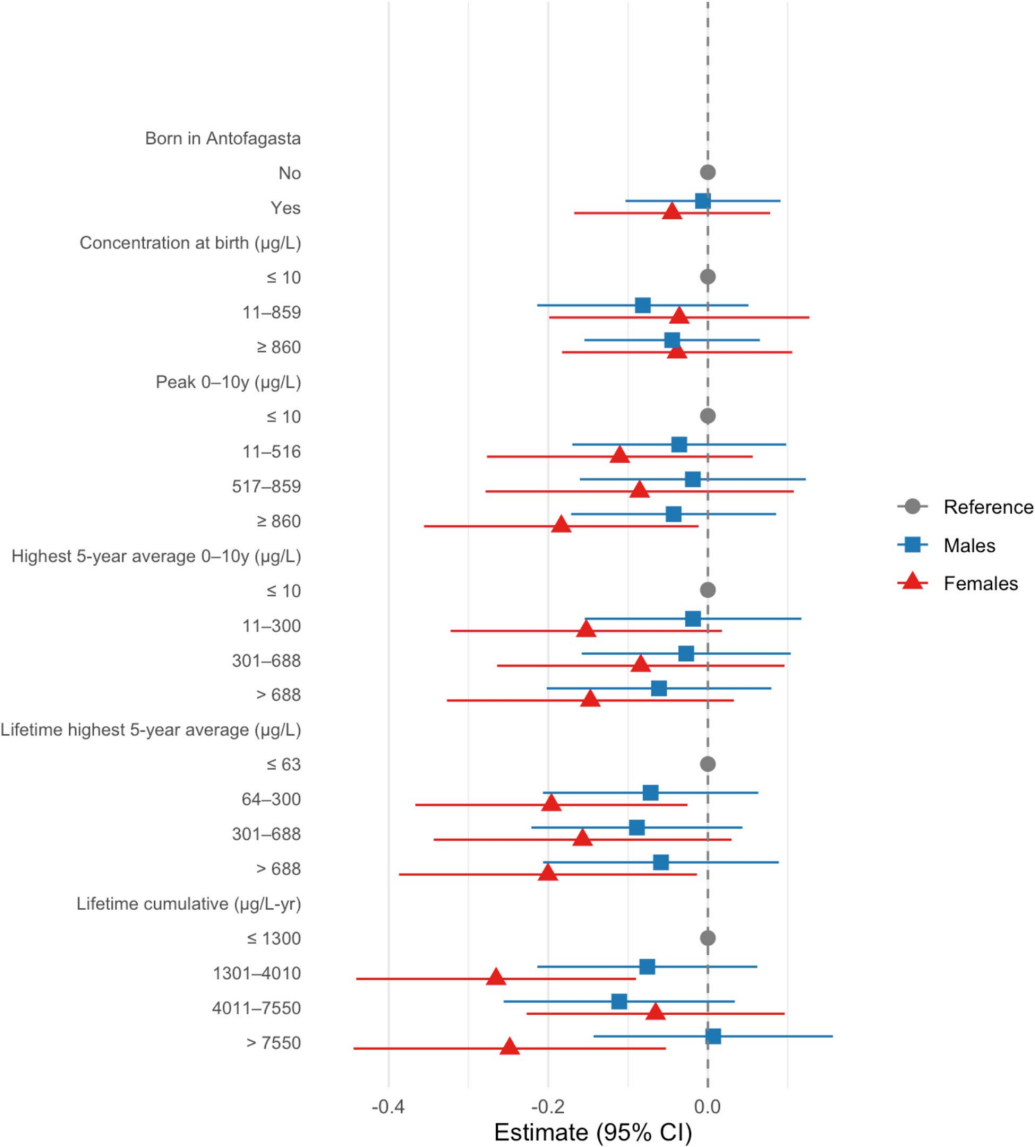
Forrest plot of regression estimates (mean difference with 95% CI) for log-transformed cortisol concentrations associated with arsenic exposure metrics stratified by sex. Exposure measures include being born in Antofagasta; arsenic concentration at birth (μg/L); peak exposure from 0 to 10 years (μg/L); highest 5-year average from 0 to 10 years (μg/L); lifetime highest 5-year average (μg/L); and lifetime cumulative exposure (μg/L-yr). Reference groups are in grey (circle) with effect estimates for males in blue (square) and females in red (triangle)

Sex-stratified analyses were also conducted to assess if associations differed between male and female participants (Fig. 2, Supplemental Table 3). Higher arsenic exposure was associated with lower plasma cortisol equivalent concentrations among both sexes. However, these effects were consistently stronger and only reached statistical significance among female participants. Specifically, female participants with a peak arsenic exposure of 860 μg/L between ages 0–10 years had mean cortisol levels that were 16.8% lower compared to participants whose highest level of exposure was ≤ 10 μg/L before age 10 (ß = −0.184; 95% CI: −0.356, −0.012). Among male participants, this association was not statistically significant (ß = −0.043; 95% CI: −0.171, 0.086). We also observed that female participants in the second (ß = −0.196; 95% CI: −0.367, −0.026) and fourth (ß = −0.200; 95% CI: −0.387, −0.014) quartiles of lifetime 5-year average arsenic exposure had approximately 18% lower mean cortisol concentrations than female participants in the first exposure quartile. Among female participants, the second (ß = −0.265; 95% CI: −0.441, −0.090) and fourth (ß = −0.248; 95% CI: −0.444, −0.053) quartiles of lifetime cumulative exposure were associated with 23.3% and 22.0% lower cortisol concentrations, respectively, than individuals in the first quartile. No statistically significant differences were observed among male participants for these exposure measures. When testing for effect modification by sex, a statistically significant interaction was observed with lifetime cumulative exposure (p-interaction = 0.036), but not for any of the other exposure measures.

We also conducted stratified analyses by age and recruitment period to assess whether these factors were driving observed associations. When stratifying results by median age (52 years), the direction of associations between arsenic exposure metrics and plasma cortisol concentrations was generally consistent across both age groups and tests for interaction by age were not statistically significant for any exposure metric (Supplemental Table 4). When stratified by recruitment period, associations between arsenic exposure metrics and cortisol concentrations were generally more negative among participants recruited in 2013 compared to those recruited in 2017 (Supplemental Table 5), although tests for interaction by recruitment year were not statistically significant.

## Discussion

This study is the first to assess whether early-life arsenic exposure has a persistent effect on plasma cortisol concentrations into adulthood in humans. Lower plasma cortisol concentrations were observed among adults exposed during early-life and these effects were observed over 40 years after exposure cessation among individuals currently living in Antofagasta, Chile—a city with a well-documented distinct period of past arsenic exposure. We also observed that associations between higher arsenic exposure and lower cortisol concentrations were stronger among female participants. Overall, this work is consistent with prior studies that show arsenic has endocrine-disrupting effects and extends these findings to include altered cortisol levels in adulthood.

This study population from Northern Chile, where high arsenic exposure was limited to the period of *in utero* and early childhood, provides a rare opportunity to investigate the long-term effects of arsenic exposure on adult cortisol levels. We observed that individuals with a peak lifetime 5-year average arsenic exposure above 63 μg/L had significantly lower morning plasma cortisol concentrations compared to those with lower arsenic exposure. Moreover, individuals in the second and fourth quartiles of lifetime cumulative arsenic exposure had lower morning plasma cortisol concentrations compared to those in the first exposure quartile, with a greater difference observed for the second quartile. These patterns suggest a non-linear relationship between arsenic exposure and cortisol concentrations, although interpretation is limited by the small sample size. Null associations were observed when using place of birth and arsenic concentration at birth, suggesting that postnatal arsenic exposure may also influence cortisol regulation later in life. These findings align with what is known about HPA axis development, which begins during gestation and continues throughout puberty [21]. Although most exposure occurred before age 10, arsenic exposure after age 10 may also affect cortisol regulation and were likely better captured by metrics that reflected both exposure intensity and duration. Together, these findings provide human evidence that chronic early-life arsenic exposure is associated with altered cortisol secretion patterns in adulthood.

Associations between lifetime cumulative arsenic exposure and lower plasma cortisol concentrations were stronger among female participants, suggesting potential sex-specific biological effects. Female participants in the second and fourth quartiles of lifetime cumulative arsenic exposure had approximately 23.3% and 22.0% lower cortisol concentrations, respectively, compared with those in the lowest exposure quartile. These cortisol differences are particularly salient when evaluated against the clinical threshold for adrenal insufficiency, defined by a morning cortisol concentration below 5.0 µg/dL [54]. Mean cortisol concentrations among female participants in the second and fourth quartiles of cumulative arsenic exposure fell below this clinical threshold (4.8 µg/dL and 4.9 µg/dL, respectively), whereas those in the lowest exposure quartile had a mean cortisol concentration of 6.3 µg/dL. In contrast, differences in morning cortisol related to arsenic exposure among male participants were minimal and not statistically significant. These findings suggest that higher lifetime cumulative arsenic exposure is associated with a shift toward adrenal insufficiency among female individuals, warranting further investigation of potential clinical implications.

Our results are consistent with the only other study that has examined the relationship between early-life arsenic exposure and cortisol levels in humans. A study of infants conducted in Arica, a city in Chile located approximately 700 km from our study population, observed lower salivary cortisol levels among high income children in the highest tertile of arsenic exposure during the second trimester of pregnancy [41]. Our study population also had generally high socioeconomic status (assessed via self-reported material items), making our study comparable to the Arica study in terms of geography, exposure timing, and socioeconomic status. However, important methodological differences limit direct comparison between the two studies. The Arica study assessed exposure by measuring total urinary inorganic arsenic (the sum of As^V^, As^III^, MMA, and DMA), which reflects recent exposure from multiple potential sources. Reported arsenic exposure levels in Arica (2.05 − 69.3 μg/L) were substantially lower than those in our study (0 − 860 μg/L), consistent with historically lower concentrations of arsenic in Arica’s drinking water [16]. Therefore, urine measurements in Salgado et al. likely reflected other sources such as soil or secondary air pollution. Additionally, the study in Arica measured salivary cortisol in children, which captures free bioactive hormone, whereas we measured plasma cortisol in adults that reflects total circulating cortisol. Despite these methodological differences, the consistent observation of lower cortisol associated with early-life arsenic exposure in both studies strengthens our findings.

In contrast to our results, a study conducted among women from West Bengal found that low-level urinary arsenic exposure (11–50 μg/L) was associated with a two-fold increase in serum cortisol, but this association was not statistically significant [42]. Some key differences between our study population and the one from West Bengal are that our study participants experienced substantially higher arsenic exposure (860 vs. 50 μg/L) and were exposed during early childhood rather than adulthood. Arsenic has a biphasic effect on the glucocorticoid receptor with lower concentrations inducing activation and higher concentrations having inhibitory effects, which might explain differences observed between studies with high versus low exposure levels [6]. Future studies should examine whether varying levels of arsenic exposure exert differential effects on the HPA axis. Null associations were also reported in other studies conducted among pregnant women and adult residents near e-waste recycling facilities in China [10, 25]. However, these studies may have been confounded by co-exposure to other heavy metals present in e-waste and the reliance on plasma arsenic concentrations, which only reflects recent exposure due to its short half-lifee in humans [4].

The observation of lower plasma cortisol concentrations 40–50 years after exposure ended suggests that arsenic has a persistent effect on HPA axis function. While the mechanisms underlying this lasting effect remain unclear, epigenetic mechanisms, such as DNA methylation of the glucocorticoid receptor (*NR3C1*), may play a role. Epidemiological studies have reported that prenatal arsenic exposure is associated with increased methylation of *NR3C1* in placental tissues [3, 9]. Additionally, trophoblasts treated with arsenic in vitro exhibited dose-dependent changes in DNA methylation at CpG sites across 12 genes involved in GR signaling, which corresponded with altered gene expression [29]. Studies have shown that epigenetic marks at the *NR3C1* locus set by early-life exposures remain stable throughout life [28, 36]. Therefore, early-life arsenic exposure may lead to lasting disruption in glucocorticoid signaling and HPA axis regulation through DNA methylation changes, though further studies are needed to confirm this mechanism. Prior animal studies have also demonstrated that developmental arsenic exposure can induce sex-specific changes in gene expression related to HPA axis signaling [1, 47]. For example, corticotropin-releasing hormone (CRH) mRNA levels were elevated in the frontal cortex of arsenic-exposed female mice but reduced in exposed male mice [47]. Glucocorticoids normally suppress CRH transcription through negative feedback mechanisms [14], but arsenic-induced CRH expression may disrupt this regulation and contribute to the more pronounced HPA axis impairment observed among female participants. Arsenic exposure also influences expression of other genes involved in glucocorticoid signaling in a sex-specific manner. In male mice, developmental exposure altered *FKBP1* and *HSD11B1* expression in ways that reduced sensitivity to glucocorticoids, potentially reflecting a compensatory mechanism not observed in female mice [47]. Lastly, stronger effects observed among female individuals could involve modulation by sex hormones, such as estradiol, which impairs glucocorticoid-dependent negative feedback of the HPA axis [55]. Future studies should examine relationships between early-life arsenic exposure, estradiol concentrations, and HPA axis regulation.

This study draws on a rare high exposure scenario in Antofagasta with clearly defined exposure onset and cessation. A key strength of this study is our ability to reconstruct accurate lifetime arsenic exposure histories using residential history. Northern Chile is the driest habitable region on Earth, with limited water sources, and most residents relied on a single municipal supply per city. Historical arsenic concentrations for these drinking water sources are available for decades, allowing us to reconstruct accurate lifetime exposure estimates based solely on residential history. Because individuals generally remember where they have lived, this approach minimizes potential recall bias. Arsenic exposure may also occur through dietary sources, such as rice and rice products, apple juice, beer and wine, and seafood [30]. However, most food in this population is imported from areas with low arsenic water concentrations since climate in the study area is so dry, further reducing exposure misclassification. While the high arsenic exposure in this study population may limit generalizability of our findings, investigating associations in populations where exposures are high, at least initially, can provide insights into the direction and magnitude of the relationship between arsenic exposure and circulating cortisol levels.

Another original aspect of this work is the application of a novel low-cost and rapid cell-based assay to measure cortisol equivalent concentrations in plasma. This method quantifies activation of the glucocorticoid receptor induced by all compounds present in plasma, providing a functional readout of total glucocorticoid activity. Serum cortisol concentrations measured using this GR bioassay were highly correlated with values obtained from a cortisol enzyme-linked immunosorbent assay [13]. Therefore, assay activity in the current study likely reflects plasma cortisol concentrations but should be confirmed using analytical techniques that specifically measure cortisol (e.g. ELISA or mass spectrometry), as other compounds present in plasma that either activate or inhibit GR could potentially cause deviations from true plasma cortisol concentrations. For instance, arsenic and its methylated metabolites have been shown to inhibit GR signaling in vitro at concentrations as low as 1 µM (~ 75 µg/L) [6, 7, 18, 23]. However, current arsenic exposure is unlikely to explain the observed reductions in cortisol equivalent plasma concentrations since annual arsenic levels in drinking water at the time of sample collection were at or below 10 µg/L (~ 133.5 nM) for all participants, which is nearly tenfold lower than the concentrations shown to inhibit GR activity in vitro. Our findings were also generally consistent with expected physiological patterns, strengthening the reliability of our cortisol measurements. Plasma cortisol equivalent concentrations in the study population ranged from 3–14 μg/dL, with a median of 6 μg/dL. These values fell within serum cortisol reference ranges of 7–25 μg/dL in the morning and 2–14 μg/dL in the evening [27]. When using spline regression to examine the relationship between time of blood draw and plasma cortisol equivalent concentrations, we observed that cortisol levels were highest among participants with collection times between 6-8AM and lower levels were observed with later collection times. This result aligns with the diurnal cycle of cortisol, which typically peaks 30 min after waking and gradually declines throughout the day [33]. Additionally, we observed that morning plasma cortisol concentrations were lower among female participants than males. This sex difference in cortisol is consistent with previous studies that reported men had higher levels of morning serum cortisol [24] and hair cortisol concentrations [49] than women.

This study had several limitations. The relatively small sample size presented a challenge given the high amount of inter-individual variability in cortisol levels [2]. Despite the limited sample size, our study was able to detect a statistically significant difference in morning plasma cortisol concentrations with arsenic exposure. For this analysis, we combined samples collected from two different collection periods, which may have potentially introduced variability due to temporal and/or batch effects. We mitigated these issues by using consistent sample processing protocols across both recruitment periods and normalization between batches using a common reference sample included on every plate. While major limitations of this study were that we did not record waking time or conduct repeat cortisol sampling to assess individual diurnal patterns, we observed the clinically expected diurnal peak at 6-8AM at the population level [27]. Since samples were collected using the same procedures in all participants, regardless of past arsenic exposure, the resulting bias would most likely be towards the null. Additionally, our results are likely not confounded by time of collection since there was no difference between arsenic exposure groups. Furthermore, studies have consistently shown high intrapersonal stability of serum cortisol [22] and our previous study using this assay found that differences between individuals were twice as large as differences within the same individual over a one-year period [13]. These data suggest that a single morning measurement can serve as a proxy of an individual's typical HPA-axis output for use in large-scale epidemiological population studies. However, future studies should collect multiple samples throughout the day to better evaluate the effect of arsenic exposure on diurnal cortisol secretion patterns. Residual confounding is also possible. Adjustments for current smoking status, education level, chronic medical conditions (hypertension, diabetes, or cancer), and technical factors (plate and recruitment period) had little impact on our results. It is also unlikely that our findings with cumulative arsenic exposure are confounded by age since plasma cortisol concentrations were not correlated with age and effect sizes changed very little with adjustment for age. Moreover, the direction of associations remained consistent in age-stratified analyses, suggesting that age is not a driver of observed associations.

In conclusion, this study is the first to provide human evidence that early-life arsenic exposure may be associated with lower HPA axis activity decades after exposure cessation. These findings contribute to growing epidemiological evidence that supports endocrine disruption as a potential mechanism underlying the long-term health effects of arsenic. Our results also suggest sex-specific susceptibility, with female individuals appearing to be more sensitive to endocrine disruption by arsenic than male individuals. Prospective longitudinal studies are needed to further characterize the arsenic exposure–response relationship and identify specific critical windows of susceptibility across the lifecourse. Futhermore, larger studies should replicate these analyses and also investigate whether cortisol dysregulation mediates relationships between early-life arsenic exposure and later development of chronic disease. Assessing endocrine-related endpoints, such as cortisol levels, is crucial both as a potential mechanism of arsenic-related disease and as an adverse health outcome with important implications for public health policies that aim to prevent arsenic exposure during early-life to mitigate long-term physiologic disruption.

## Supplementary Information

Supplementary Material 1.

## Data Availability

The datasets used for the current study are available from the corresponding author upon reasonable request.
